# Intraspecific rearrangement of mitochondrial genome suggests the prevalence of the tandem duplication-random loss (TDLR) mechanism in *Quasipaa boulengeri*

**DOI:** 10.1186/s12864-016-3309-7

**Published:** 2016-11-24

**Authors:** Yun Xia, Yuchi Zheng, Robert W. Murphy, Xiaomao Zeng

**Affiliations:** 1 Chengdu Institute of Biology, Chinese Academy of Sciences, Chengdu, 610041 China; 2 Centre for Biodiversity, Royal Ontario Museum, 100 Queen’s Park, Toronto, ON M5S 2C6 Canada

**Keywords:** Mitochondrial gene order, tRNA, Mitogenomics, Intermediate mitogenomic rearrangement, Random gene loss

## Abstract

**Background:**

Tandem duplication followed by random loss (TDRL) is the most frequently invoked model to explain the diversity of gene rearrangements in metazoan mitogenomes. The initial stages of gene rearrangement are difficult to observe in nature, which limits our understanding of incipient duplication events and the subsequent process of random loss. Intraspecific gene reorganizations may represent intermediate states, and if so they potentially shed light on the evolutionary dynamics of TDRL.

**Results:**

Nucleotide sequences in a hotspot of gene-rearrangement in 28 populations of a single species of frog, *Quasipaa boulengeri*, provide such predicted intermediate states. Gene order and phylogenetic analyses support a single tandem duplication event and a step-by-step process of random loss. Intraspecific gene rearrangements are not commonly found through comparison of all mitochondrial DNA records of amphibians and squamate reptiles in GenBank.

**Conclusions:**

The intraspecific variation in *Q. boulengeri* provides insights into the rate of partial duplications and deletions within a mitogenome, and reveals that fixation and gene-distribution in mitogenomic reorganization is likely non-adaptive.

**Electronic supplementary material:**

The online version of this article (doi:10.1186/s12864-016-3309-7) contains supplementary material, which is available to authorized users.

## Background

The order of mitochondrial (mt) genes in metazoans varies greatly [1, 2] and the molecular drivers that explain the underlying evolution are subject to debate [3, 4]. The most widely invoked model involves tandem duplication of mt genes followed by the random loss of one copy (TDRL) [5–8]. However, duplication and non-random loss may result from the transcriptional polarities of genes and their positions in the genome, as two models describe: tandem duplication and non-random loss (TDNL) [9]; and dimer-mitogenome and non-random loss (DMNL) [10]. These models attribute gene-rearrangements to clustering by common polarity. Further gene rearrangement may be a result of intra- or inter-mtDNA recombination [11, 12]. Recently, Shi et al. [13] proposed that double replications and random loss (DRRL) best explained gene rearrangements in flounders.

The TDRL model is most widely accepted, but no definitive evidence supports it. According to this model, tandem duplications involve imprecise terminations, strand slippages, and/or mispairings, which result in errors in mtDNA replication [14, 15]. The hypothesis predicts that the new, intermediate mitogenome will contain duplicated genes, one of which is subsequently randomly lost. Despite the increasing number of taxa known to have gene rearrangements, few mitogenomes exhibit intermediate states that could point to this evolutionary mechanism, even though pseudogenes and residues of tandemly duplicated sequences may provide indirect evidence for an intermediate step in genomic rearrangement [3, 6, 16].

If gene deletion occurs randomly, then populations should have mitogenomes with varying gene-orders that consist of alternative arrangements of duplicated genes. Such alternative gene arrangements have been reported in only a few closely-related lineages or species [16, 17]. These interspecific occurrences support the TDRL model, yet no information exists as to when and where the rearrangements occurred and how they subsequently dispersed within a species. Investigations at a lower (intraspecific) level may be necessary to understand the evolution of mitochondrial gene rearrangements.

Intraspecific rearrangements of mitogenomes are rarely reported in vertebrates. Many species have structural diversity in their control region (CR), but all of these involve non-coding sequences. Gene order diversity within a species is known only from asexual squamates [7, 14, 18], an amphisbaenid [19] and a bird [20]. In these cases, gene rearrangements that qualify as potential intermediate states involve either a large number of genes adjacent to the CR or the formation of pseudogenes [3, 19]. In addition, gene rearrangements in the mitogenomes of asexual squamates results from multiple independent duplications and lack the random loss of alternative states [7].

**Fig. 1:**
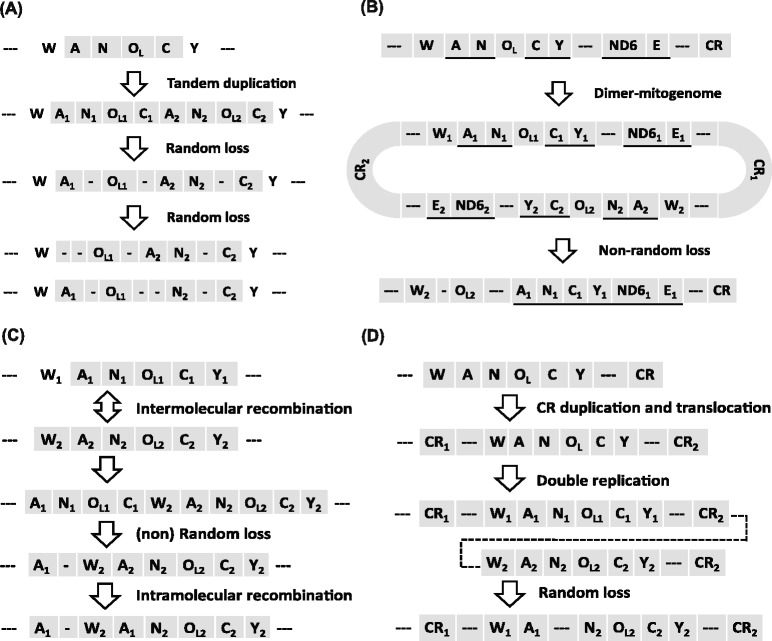
Expected mitochondrial gene rearrangement under different evolutionary scenarios. **a** Tandem duplication–random loss model (TDRL): A, N, O_L_, C were tandem duplicated, followed by random loss of the redundant copies. Random loss could occur repeatly, resulting in alternative loss types [5]. **b** Tandem duplication and non-random loss (TDNL), or dimer-mitogenome and non-random loss (DMNL) models: a dimeric molecule was formed by two monomers linked head-to-tail, then one of the two sets of promoters lost function, and genes with the same polarity would cluster together [9, 10]. **c** Inter- or intra-mtDNA recombination: duplication was caused by unequal crossing over of intermolecular recombination. Redundant copies were then deleted. Intramolecular recombination could cause concerted evolution of the two copies of *trnA* [12]. **d** Double replications and random loss (DRRL) model: the CR was duplicated and translocated, then double replications of the mitogenome were successively initiated from the two CRs, leading to the duplication of the genes between the two CRs, followed by random loss [13]. Underline indicates the transcriptional direction of L-strand–encoding gene. “---” represents other coding gene. “-”, pseudogenes or noncoding sequence. Gray boxes represent the genes involved in rearrangement

High levels of gene rearrangement have been reported from amphibians, especially among so-called modern frogs [21–23]. A hotspot of gene rearrangement has been reported in the “WANCY” region (*trnW*, *trnA*, *trnN*, origin of light strand replication (O_L_), *trnC*, and *trnY*) [6, 24]. Because many amphibians have gene rearrangements in their WANCY region [17, 25, 26], reorganizations in this group facilitate testing hypotheses on how gene-rearrangement occurs. Each of the above hypotheses predicts a unique arrangement of five short tRNA genes in the WANCY region (Fig. 1), which can be compared with the results of sequencing.

Herein, we report a diversity of rearrangements in the frog *Quasipaa boulengeri* and the discovery of intermediate states. To test the hypotheses of mechanisms of gene rearrangement, we investigate the origins and evolution of the rearrangement by 1) determing the structure of the rearranged region for each type, 2) speculating on the steps resulting in observed gene rearrangements, and 3) placing each type on an inferred phylogeny and estimating the time at which each rearrangement arose. We supplement this with an *in silico* approach using mitochondrial gene orders from GenBank data. By using a custom Perl script (mtGordV0.5.pl), we explore the frequency of occurrence of intraspecific rearrangements in Amphibia and Squamata, which have high diversities in mitogenomic rearrangements.

## Results and Discussion

### Intraspecific rearrangements

**Fig. 2:**
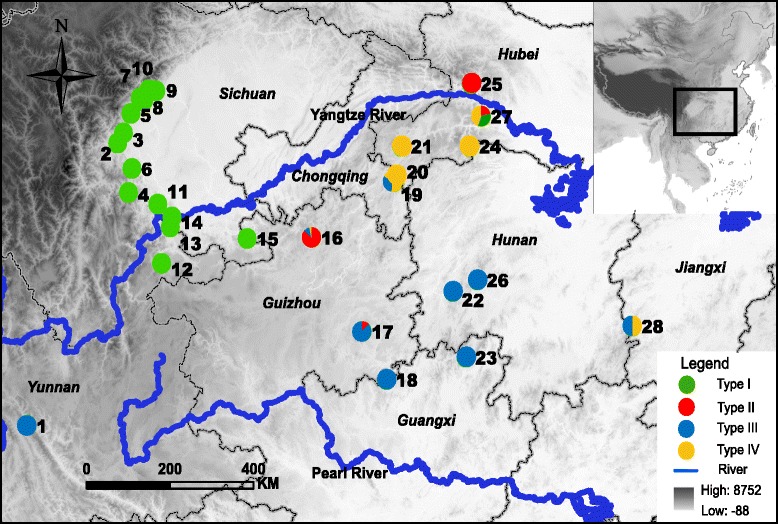
Map of sampling localities for *Quasipaa boulengeri*. Populations are presented as pie-diagrams with slice-size proportional to the frequency of type of mitochondrial gene rearrangement. Green: Type I; red: Type II; blue: Type III; yellow: Type IV. This map is created with ArcGIS (ESRI, http://www.esri.com/software/arcgis)

We obtained 290 samples from 28 localities for *Quasipaa boulengeri* (Fig. 2 and Additional file 1: Table S1). Sequences from a region encompassing *nad2* to *cox1*, which includes the WANCY hotspot, revealed gene-organizations atypical of vertebrates. Stable secondary structures of tRNA genes and the absence of premature stop codons in *nad2* and *cox1* authenticated the sequencing of mtDNA. The WANCY region in this frog differed from the typical organization by having a long noncoding sequence that ranges in size from 473 bp to 925 bp. Further, gene annotation identified different positions for the gene and its copy, even within a single population. The details of gene organization of the WANCY region for each sample were listed in Additional file 2: Table S2.

**Fig. 3:**
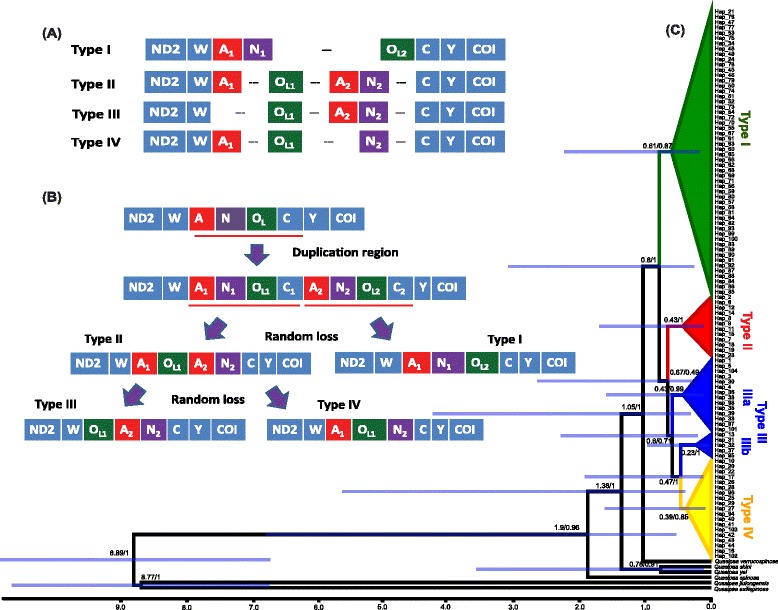
Diversity of intraspecific mitochondrial gene rearrangements in *Quasipaa boulengeri*. **a** Four types of gene rearrangement in the “WANCY” region. **b** Types of evolution and putative mechanism of gene rearrangement of the mitochondrial sequences according to the tandem duplication–random loss model (TDRL). TDRL first produces Types I and II. Type II is the intermediate state with two *trnA* genes. Types III and IV result from the random loss of one alternative *trnA*. “---”, pseudogenes or noncoding sequence. **c** Phylogenetic relationships and divergence times of four mitochondrial gene rearrangements in *Quasipaa boulengeri*. Tree topology derived from BI analyses of *cox1* and *cob* is consistent with an ML tree. Numbers above the lines or beside the nodes are inferred divergence times (Ma) and Bayesian posterior probabilities, respectively. Types II, III, and IV form a clade, and each Type forms its own clade, except for Type III

Annotation identifies four kinds (types) of gene rearrangements (Fig. 3a). The typical gene order of the *trnW*–*trnY* block is *trnW*, *trnA*, *trnN*, O_L_, *trnC*, and *trnY* (WANCY). Unlike the other types, where the O_L_ is located before *trnN* (Fig. 3a), in Type I, the O_L_ occurs after *trnN*, separated by an intergenic spacer (IGS or noncoding region). In Type II, two *trnA* occur with an IGS located between *trnA1* and O_L_, another IGS occurs between O_L_ and *trnA2*, and another IGS between *trnN* and *trnC*. The gene organization of Type III and Type IV are similar to Type II, but Type III lacks *trnA1* and Type IV lacks *trnA2*, respectively. Except for the reorganizations of *trnA*, *trnN*, and O_L_, all other tRNAs and protein-coding genes have positions and lengths typical of the vertebrate mitogenome (Fig. 3a; Additional file 2: Table S2).

### Sequential process of TDRL

All four types of gene rearrangement in *Q. boulengeri* involve *trnA*, *trnN*, O_L_ and *trnC*. The IGSs reveal tandem duplications in the WANCY region. These residues identify pseudogenes of *trnA*, *trnN* and *trnC*, whose sequences are similar to corresponding tRNAs. Additional file 3: Figure S1 shows the primary sequence of *trnA* and *trnN* for each variant. The secondary structures of these genes fold into typical stem-and-loop structures (Additional file 4: Figure S2). Each type of variant has only one *trnA* and *trnN*, except in Type II, which has two *trnA*s, both of which form stable clover-leaf structures. Thus, these genes are paralogs created by gene duplication. Residues are very similar to *trnA* and *trnN*, but have a loss of function owing to secondary structures or a mutation on the anticodon position.

TDRL [5, 16] best explains the gene rearrangement in *Q. boulengeri*, and the diversity of rearrangements rejects the alternative hypotheses of non-random loss (TDNL and DMNL), recombination, and DRRL. Genes of the same polarity do not cluster together, and the finding of alternative loss of duplicated genes, as seen in comparison of Types III and IV, contradicts non-random loss models (TDNL or DMNL). The absence of different tandem duplication junction points and no variation in the number of tandem repeats (VNTR) and this does not support the recombination model. We cannot reject the hypotheses that unequal crossing over of intermolecular recombinations were subtly inserted in front of *trnA* and behind *trnC*, but tandem duplication would essentially be a consequence of this recombination. Further, no concerted evolution rejects intramolecular recombination, because the two copies of *trnA* in type II differ (Additional file 3: Figure S1, and Additional file 4: Figure S2). Finally, analyses reject the hypothesis of DRRL due to the absence of two control regions in the mitogenome of *Q. boulengeri* [26].

The TDRL hypothesis remains the only viable explanation and our results conform to its predictions (Fig. 3b). The hypothesized duplicated region in the mitogenome of *Q. boulengeri* includes *trnA*, *trnN*, O_L_, and a partial fragment of *trnC*. Slipped-strand mispairing, imprecise termination or recombination have been proposed to explain mitogenomic duplications [5, 7, 15]. Regardless of the molecular mechanism, tandem duplication mutations will yield two copies each of *trnA*, *trnN*, O_L_, and *trnC*. Subsequent random loss appears to have occurred at least twice independently in the mitogenome of *Q. boulengeri*. First, rearrangement Type I involves the loss of O_L1_, *trnC1*, *trnA2*, and *trnN*2. Second, loss involves *trnN1*, *trnC1*, and O_L2_ in Type II. The retention of two copies of *trnA* in Type II is direct evidence for the randomness of loss because alternative losses occur in Type III and Type IV, which have the same gene order as Type II (*trnA1* has been lost in Type III as compared to *trnA2* in Type IV). Rather than a result of selection for one or other alternative, loss of one copy of *trnA* appears to have occurred by chance alone.

The sequencing *cox1* and *cob* for 290 individuals (Additional file 1: Table S1) identifies the origin of the initial tandem duplication event and the stepwise process of random loss when viewed in terms of the phylogenetic relationships of all types of gene rearrangement. The concatenated alignment contains 1463 nucleotide positions (*cox1*: 626 bp; *cob*: 837 bp) without stop codons. Maximum likelihood (ML) and Bayesian inference (BI) reconstructions obtain similar tree topologies for the four types of rearrangement (Fig. 3c). All haplotypes cluster together by type and with moderate to strong levels of nodal support, except for Type III, which is paraphyletic. Analyses recover the group Type II + Type III + Type IV, and roots it as the sister-group of Type I. Type II forms the sister-group of Type III + Type IV and some samples of Type III unite with Type IV.

The phylogenetic analyses and gene-order strongly indicate a single tandem duplication event and stepwise random loss in *Q. boulengeri*. The clustering of Types II–IV suggests that they share the same primary TDRL process (Fig. 3b, c), and that Type I represents an independent random loss. The monophyly of types I, II and IV indicate a single origin of each rearrangement type. Paraphyly of Type III suggests two parallel random losses are responsible for the same gene rearrangement. The associations of Type IIIa, IIIb and IV indicate that they shared a recent common ancestor, but independently lost one duplicated copy of *trnA*. The possession of both copies of *trnA* indicates that Type II represents the intermediate state.

Although mitochondrial gene rearrangements are not uncommon among related taxa, recognized intermediate steps of gene-order rearrangements are rare, and their presence can suggest evolutionary mechanisms [7, 27]. Most intermediate states appear as pseudogenes or residues of tandemly duplicated sequences rather than as two functional gene copies [3, 8, 16]. The intermediate state of Type II, leading either to Type III or Type IV, is an example of the random loss of one *trnA* gene.

Alternative loss-types have been observed among closely related species, e.g. alternative losses of *trnH* in the anuran *Babina* [17, 28]. However, alternative losses have been reported rarely within a single species and even more so within a population. The finding of alternate losses in *Q. boulengeri* is the first observation of an intermediate state involving two functional gene copies, and, simultaneously, the loss of alternative types in a vertebrate mt genome.

The occurrence of TDRL in *Q. boulengeri* corresponds to the view that reorganization of the mitochondrial genome is nonadaptive [17, 29]. Our results indicate that the hotspot of gene rearrangement is adjacent to the origin of light-strand replication [6]. Homoplastic mitochondrial rearrangements are contiguous in the genome or they locate around the origin of replication [6, 30, 31]. Under these conditions, mitochondrial gene-orders appear susceptible to convergent or parallel evolution because of functional constraints or selective pressures [32–34]. However, such evolution does not occur in *Q. boulengeri*. The gene-order and phylogenetic analyses indicate a single tandem duplication event followed by independent losses (Fig. 3), which implies that random loss and gene-order are not involved in adaptive evolution [17].

### Evolution of mitogenomic rearrangement

A time-calibrated phylogeny constructed using Bayesian inference estimates the recency of mitogenomic rearrangements in *Q. boulengeri* (Fig. 3c). The initial diversification (Type I) dates to about 0.8 Ma and divergence among the other three types ranges from 0.4 to 0.6 Ma, suggesting that the duplication and fixation of these rearrangements can occur quite quickly. Combing interspecific rearrangement data, we summarize the rates of mitogenomic duplication and loss (Additional file 5: Table S3). This suggests that post duplication, the alternative loss types can occur in 0.2–5 Ma.

To explore how many intraspecific rearrangements existed, we gather both *in silico* and experimental evidence to detect the gene order in highly rearranged groups. First, our *in silico* approach obtains mitochondrial gene-order information (mtGordV0.5.pl;  Additional file 6: Software) from GenBank in Amphibia and Squamata, two groups with high diversities in mitogenome rearrangement. Analyses discover that intraspecific rearrangements are rare, but they exist. Two cases occur in Squamata, one in asexual squamates with multiple origins of duplication [7], and another in an amphisbaenian with alternative loss-types varying among populations [19]. No intraspecific rearrangements in Amphibia exist in data from GenBank.

Our sequences of the WANCY fragment in multiple populations of the frogs *Odorrana schmackeri* and *Amolops mantzorum* detected that this hotspot region differed from the typical vertebrate arrangement [17]. These species do not exhibit intraspecific variation in gene-order, yet evidence for variation in losses may be represented by the residues of pseudogenes (data not shown).

Intraspecific studies may provide new insights into the high incidence of rearranged mitochondrial genomes. Above the species level, rates of mitogenomic partial duplication have been found to be high, and multiple duplication events can facilitate gene-rearrangement [7, 16, 21]. However, duplications may not occur frequently within a species. The rarity of this may reflect selection or functional constraints that prevent fixation, and may shed light on the limits of intraspecific diversity of mitogenomes. It could also owe to the dearth of investigations of intraspecific mitogenomic reorganization. We predict that mitochondrial metagenomic skimming by next-generation sequencing [35, 36] will detect additional cases of intraspecific rearrangements.

Random loss within duplicated regions could occur repeatedly, and the rate of duplication excision may be relatively high. At the intraspecific level, random loss occurred independently many times both in *Q. boulengeri* and the lizard *Bipes biporus* (Fig. 3c, Additional file 5: Table S3). At the interspecific level, the sibling frog genera *Babina* and *Odorrana* share the same duplication of genes, but the pathways of deletion differed among species [17, 28].

The loss of a duplicated region and fixation could occur in short evolutionary time (0.2 Ma, Additional file 5: Table S3). Deletion of a redundant gene-copy may happen rapidly due to functional constraints and the compactness of the metazoan mitogenome, facilitating the formation of pseudogenes or the complete deletion of redundant genes [27, 37]. A functionally redundant duplicate gene copy may not persist long in a population because deleterious mutations can accumulate and cause the redundant gene to become nonfunctional [38].

Nonadaptive forces, such as genetic drift or bottlenecking, may drive the fixation and dispersal of mitogenomic reorganizations. The low effective population size of the mitogenome leaves it vulnerable to bottlenecks and genetic drift, which can drive the fixation of large-scale genomic modifications [16, 39–41]. *Quasipaa boulengeri* resides in localized montane areas, mainly in rocky streams [42]. Its highly specific habitat may limit gene flow and result in a pattern structured by genetic drift. The upper and midstream tributaries of the Yangtze River, including some areas in Chongqing, Guizhou and Hubei, have populations containing two or more sympatric types of rearrangements (Fig. 2). This area may be the original source of the gene rearrangements, or may represent areas of secondary contact. Both scenarios explain the distribution of types. Historical demographic analyses in this area point to dispersal events for *Q. boulengeri* [42]. TDRL may have first occurred in this area, followed by dispersal to other places. However, the single origin of each type suggests independent fixations of alternative arrangements, in which case secondary contact could also explain the pattern.

## Conclusion

The initial stages of gene rearrangement are difficult to observe in nature, which limits our understanding of the evolutionary mechanism. Intraspecific or population level investigations may represent intermediate states and fixation of initial rearrangement, and if so they potentially shed light on the evolutionary dynamics. Here, we found mitogenomic rearrangements diversity in a single frog species, *Quasipaa boulengeri*. Intermediate state and alternative losses types were observed in this frog, which provide direct evidence of tandem duplication and random loss model for mitochondrial gene rearrangement. The intraspecific variation in *Q. boulengeri* provides insights into the rate of partial duplications and deletions within a mitogenome, and reveals that fixation and gene-distribution in mitogenomic reorganization is likely non-adaptive. Our observation may shed light on the investigations of intraspecific mitogenomic reorganization.

## Methods

### Samples and Sequence Amplification

A total of 290 samples from 28 localities were used. Frogs were collected from 2006 to 2013, and Fig. 1 and Additional file 1: Table S1 detail the localities. Tissue samples, including liver, muscle, and tadpoles were stored in 95% ethanol at −20 °C in the Chengdu Institute of Biology, Chinese Academy of Sciences (CIB). The CIB Animal Care and Use Committee approved all procedures.

We sequenced the hotspot of gene rearrangement, from *nad2* to *cox1*, of mtDNA for *Quasipaa boulengeri*, which included the WANCY region [26]. Two other fragments, *cox1* and *cob*, were sequenced for population genetic and phylogenetic analysis. For *cox1*, we added published sequences (GenBank No. JX629572–JX629667) for phylogenetic analysis [43]. The sample and sequence information were provided in Additional file 1: Table S1. The PCR primers for the three fragments were those of Kurabayashi and Sumida [44] and Qing et al. [43]. To avoid Numt (nuclear copies of mtDNA genes), we designed a pair of primers to confirm amplification of the WANCY region: 5059 F-3, 5’- TTCTTTTACTTACGACTGACAT -3’; 6399R-2, 5’- ATGCCTGCGGCTAAAACTGGAAGAG-3’. PCR amplification, sub-cloning, and sequencing followed Xia et al. [17]. All newly obtained sequences were examined by checking for the presence of premature stop codons (pseudogenes).

### Gene Annotation and Time-Tree Analyses

The tRNA genes were identified by using both tRNAscan-SE v.1.21 (http://lowelab.ucsc.edu/tRNAscan-SE) and MITOS (http://mitos.bioinf.uni-leipzig.de). To avoid misannotated tRNA genes, we predicted the secondary structure for each. We extracted and aligned the duplicated tRNA genes and their pseudogene residues.

We aligned sequences of each fragments using ClustalW in MEGA6 [45]. DnaSP v.5.10 [46] was used to determine DNA polymorphisms and divergences. To estimate the time-tree, we constructed phylogenies using *cox1* and *cob*, and partitioned these genes by codon position. Six species of *Quasipaa*, including *Q. verrucospinosa* (KF199147), *Q. shini* (KF199148), *Q. yei* (KJ842105), *Q. spinosa* (FJ432700), *Q. jiulongensis* (KF199149) and *Q. exilispinosa* (KF199151), were chosen as outgroup taxa. The best-fit substitution model for each partition was estimated using the Akaike information criterion (AIC) implemented in PartitionFinder v1.1.1 [47]. The best model of each partition was chosen for maximum likelihood (ML) and Bayesian inference (BI) analyses, which were performed with RAxML BlackBox web-servers (http://phylobench.vital-it.ch/raxml-bb/index.php) [48] and MrBayes v.3.1 [49], respectively.

BI as implemented in BEAST2 v.2.1.2 [50] was used to obtain an ultrametric time-tree for *Q. boulengeri*. Each locus was assigned its own partition with unlinked substitution model but with linked clock and tree models. We assumed a substitution rate ranging from 0.65 to 1.00% per Ma for the *cox1* and *cob* based on evolutionary rates commonly proposed for frogs [42, 51, 52]. Lacking fossil evidence, we calibrated our phylogeny using the published divergence time to the most recent common ancestor (TMRCA) between the *Q. jiulongensis* and *Q. exilispinosa* of about 9 Ma [53]. We ran BEAST for 20 million generations while logging trees every 1000 generations for a total of 20,000 trees. We determined a 10% burn-in length using Tracer v.1.5 and retained the maximum clade credibility tree using TreeAnnotator v.2.1.2.

A Perl script named mtGordV0.5.pl was written by YZ to obtain the gene-orders of mitochondrial records deposited in GenBank, based on the annotation of the sequence. Records were downloaded together as a single file, which was used as the input file of the script. For each record with more than one gene, items in the order of accession number, sequence length, species name, gene names in their original order, and total number of genes were saved in an individual line to the output file. Items were separated from each other by a tab. The script was applied to two major groups of vertebrates, amphibians and squamate reptiles. For amphibians, all 126,638 mitochondrial records were downloaded on 02 Nov 2015, and the output file contained 17,559 records. For squamates, all 110,064 records were downloaded on 25 Sep 2015, and the output file contained 21,045 records. The output files were opened using Microsoft Excel and records were aligned according to species names. The records were manually checked for intraspecific and intrageneric cases of random loss of genes after duplication. As the script did not include all variation of annotations for all mitochondrial genes, errors from missing genes were expected for a small number of records. However, when a potential case was detected, the related GenBank full records were carefully checked. More importantly, this script made such a scan possible, analyses could be conducted within a reasonable amount of time, a few days for each group in our case, and it could be applied to other groups of taxa. Regarding the speed of the script itself, the data for squamates were processed within 3 min on a ThinkPad X200 laptop computer.

## Additional files

Additional file 1: Table S1. Summary of sample localities and mitogenome regions sequenced for *Quasipaa boulengeri*. (XLS 84 kb)
Additional file 2: Table S2. The gene organization of “WANCY” region for each sample. (XLS 77 kb)
Additional file 3: Table S3. Time-scale of mitogenomic duplication and radom loss. (PDF 209 kb)
Additional file 4: Figure S1. The primary sequence of *trnA* and *trnN* for each variant. (PDF 161 kb)
Additional file 5: Figure S2. The secondary structures of of *trnA* and *trnN* fold into typical stem-and-loop structures. (DOCX 22 kb)
Additional file 6: Software. The Perl script mtGordV0.5.pl with its readme file and example input and output files. (ZIP 5.35 kb)

## Abbreviations

BI: Bayesian inference
*cob*: Cytochrome b
*cox1*: Cytochrome c oxidase subunit I
CR: Control region
DMNL: Dimer-mitogenome and non-random loss
DRRL: Double replications and random loss
IGS: Intergenic spacer
ML: Maximum likelihood
*nad2*: NADH dehydrogenase subunit 2
O_L_: Origin of light strand replication
TDNL: Tandem duplication and non-random loss
TDRL: Tandem duplication and random loss
WANCY: *trnW*, *trnA*, *trnN*, O_L_, *trnC*, and *trnY*
