# Supplementary material for: Intraspecific rearrangement of mitochondrial genome suggests the prevalence of the tandem duplication-random loss (TDLR) mechanism in Quasipaa boulengeri
Source: BMC Genomics. 2016 Nov 24;17:965. doi: 10.1186/s12864-016-3309-7 (PMC5122201; doi:10.1186/s12864-016-3309-7)

Supplementary Figure S1

Sequence of *trnA*

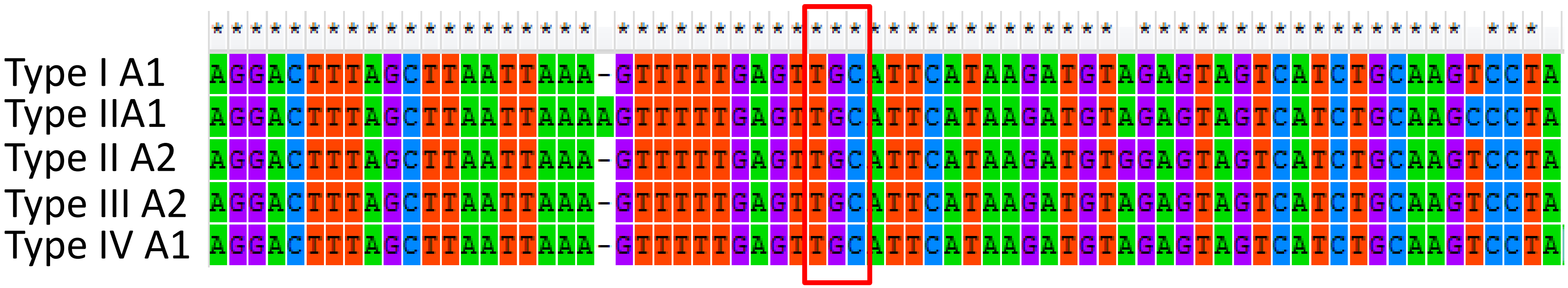

Sequence of *trnN*

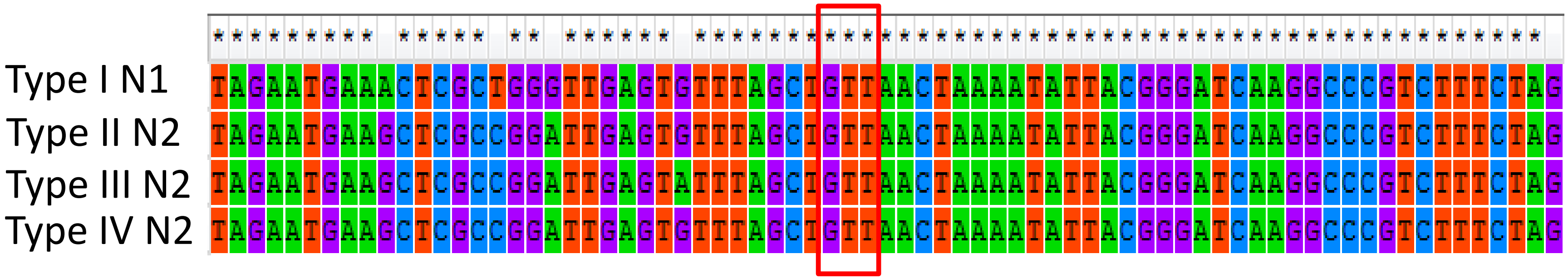

Supplement: Additional file 3: Table S3. — Time-scale of mitogenomic duplication and radom loss. (PDF 209 kb) [file 12864_2016_3309_MOESM3_ESM.pdf]
