# Supplementary material for: Intraspecific rearrangement of mitochondrial genome suggests the prevalence of the tandem duplication-random loss (TDLR) mechanism in Quasipaa boulengeri
Source: BMC Genomics. 2016 Nov 24;17:965. doi: 10.1186/s12864-016-3309-7 (PMC5122201; doi:10.1186/s12864-016-3309-7)
Supplement: Additional file 4: Figure S1. — The primary sequence of trnA and trnN for each variant. (PDF 161 kb) [file 12864_2016_3309_MOESM4_ESM.pdf]

Supplementary Figure S2-a

Predicted secondary structures for *trnA*

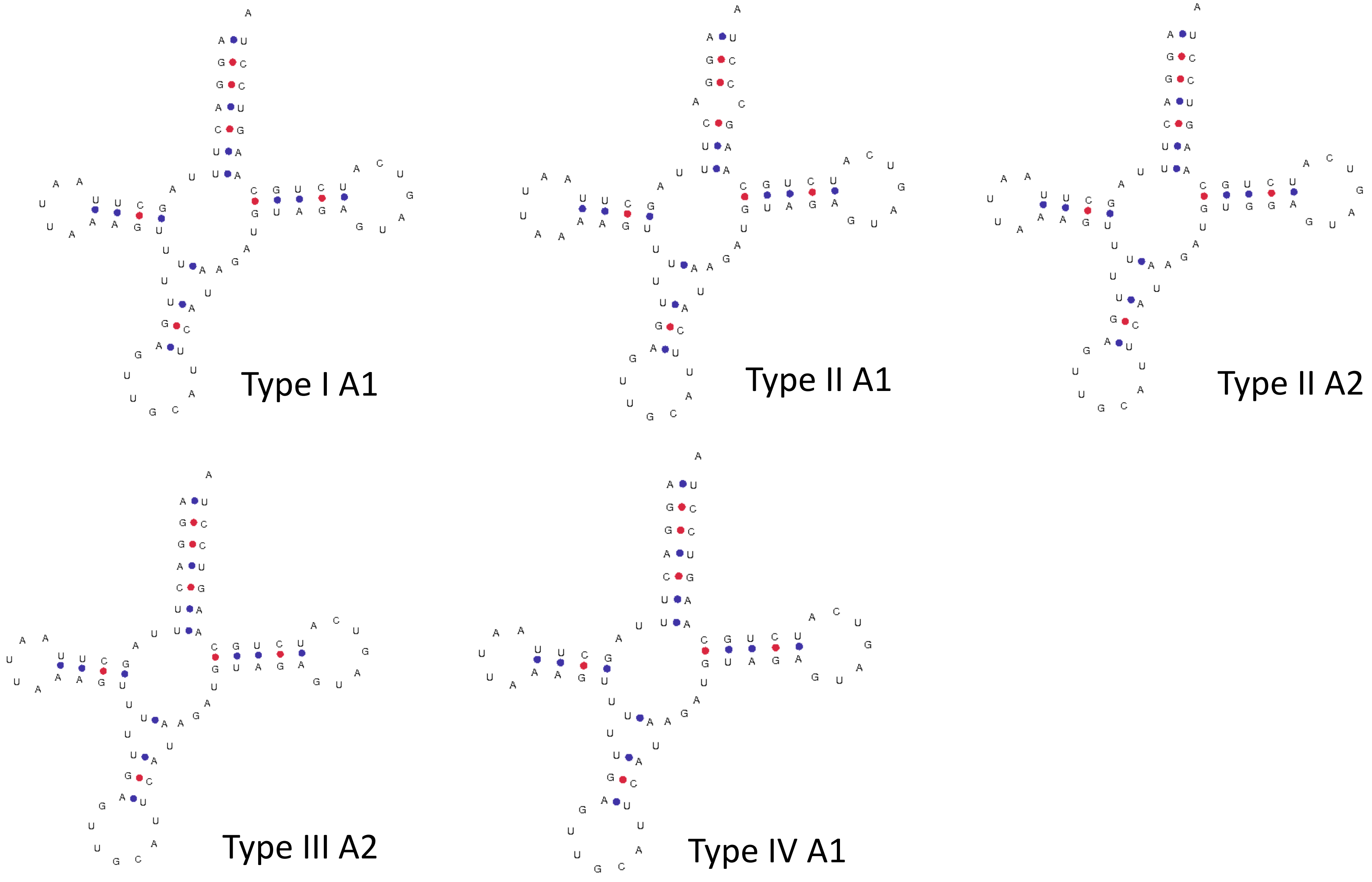

Supplementary Figure S2-b

Predicted secondary structures for *trnN*

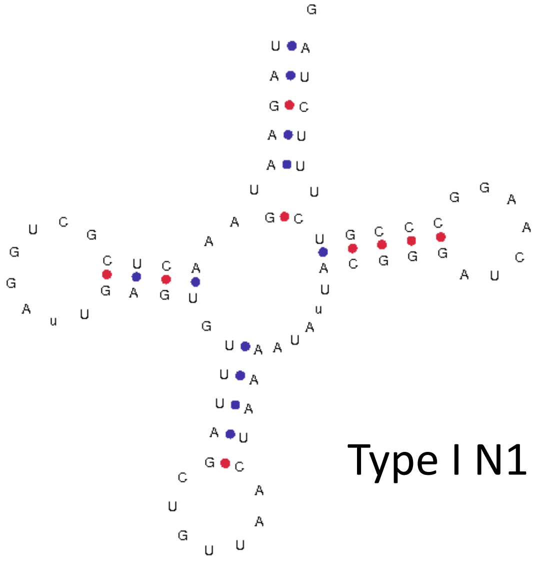

Type I N1

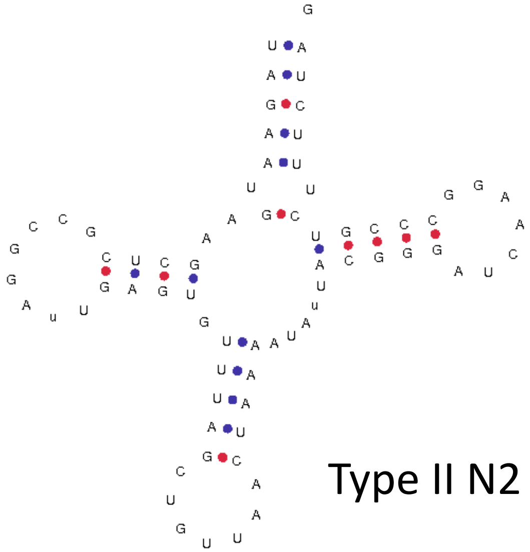

Type II N2

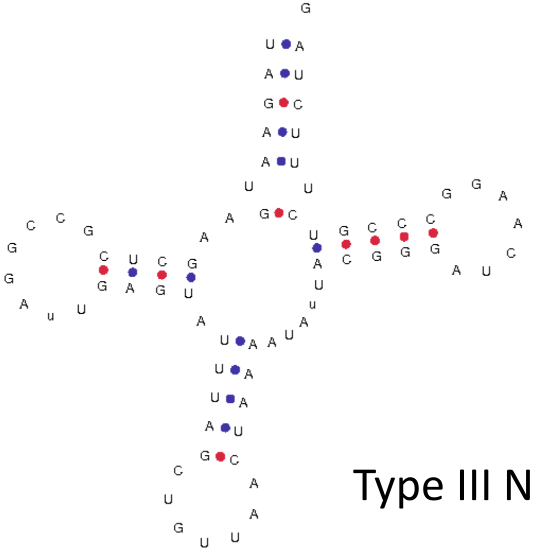

Type III N2

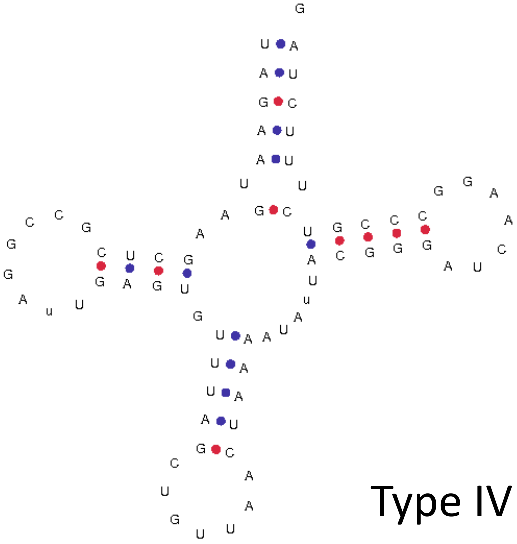

Type IV N2
