# Supplementary material for: Intraspecific rearrangement of mitochondrial genome suggests the prevalence of the tandem duplication-random loss (TDLR) mechanism in Quasipaa boulengeri
Source: BMC Genomics. 2016 Nov 24;17:965. doi: 10.1186/s12864-016-3309-7 (PMC5122201; doi:10.1186/s12864-016-3309-7)
Supplement: Additional file 5: Figure S2. — The secondary structures of of trnA and trnN fold into typical stem-and-loop structures. (DOCX 22 kb) [file 12864_2016_3309_MOESM5_ESM.docx]

Supplementary Table S3 Time-scale of mitogenomic duplication and radom loss.

| Species or genus | Rearrangement type I | Rearrangement type II | Rearrangement type III | Rearrangement type IV | Time (Mya) | References | |
| --- | --- | --- | --- | --- | --- | --- | --- |
|  |  |  |  |  |  | Mitogenomic rearrangement | Divergence time |
| *Quasipaa boulengeri* | ND2–W–A_1_–N_1_–O_L2_–C–Y–COI | ND2–W–A_1_–O_L1_–A_2_–N_2_–C–Y–COI | ND2–W–O_L1_–A_2_–N_2_–C–Y–COI | ND2–W–A_1_–O_L1_–N_2_–C–Y–COI | 0.2–1 | This study | This study |
| *Bipes biporus* | Cytb–T_1_–P_1_–P_2_–CR (AF013251) | Cytb–T_1_–P_2_–CR (AY605481) | Cytb–T_2_–P_2_–CR (AY605480) |  | 1–5* | Macey et al. 1998 Macey et al. 2004 | Brandley et al. 2011 |
| *Babina* & *Odorrana* | ND4–H–S_1_–ND5–ND6–E–Cytb–CR–L_2_ (AB761266) | ND4–H–ND6–Cytb–CR–S_1_–ND5–E–L_2_ (AB761264) | ND4–S_1_–ND5 (KF771303) | ND4–S_1_–ND5–ND6–E–Cytb–CR–H–L_2_ (AB511282) | 2–5 | Kakehashi et al. 2013; Xia et al. 2014 | Chen et al. 2013; Li et al. 2012 |
| *Phrynocephalus versicolor & Phrynocephalus przewalskii* | Cytb–T–CR–F–P–CR–F–CR–12S (KJ749841) | Cytb–T–CR–F–CR–F–P–CR–12S (KF572032) |  |  | 3.34 | Li et al. 2015; Song et al 2016 | Guo & Wang 2007 |

* The divergence time of each rearrangement type in *Bipes biporus* was calculated by substitution rate (0.5% per Myr for Cytb).

**References**

Brandley MC, Wang Y, Guo X, Montes de Oca AN, Fería-Ortíz M, Hikida T, Ota H. 2011. Accommodating Heterogenous Rates of Evolution in Molecular Divergence Dating Methods: An Example Using Intercontinental Dispersal of Plestiodon (Eumeces) Lizards. *Syst Biol* 60:3-15.

Chen X, Chen Z, Jiang J, Qiao L, Lu Y, Zhou K, Zheng G, Zhai X, Liu J. 2013. Molecular phylogeny and diversification of the genus Odorrana (Amphibia, Anura, Ranidae) inferred from two mitochondrial genes. *Mol Phylogenet Evol* 69:1196–1202.

Guo XG, Wang YZ. 2007. Partitioned Bayesian analyses, dispersal-vicariance analysis, and the biogeography of Chinese toad-headed lizards (Agamidae : Phrynocephalus): A re-evaluation. *Mol Phylogenet Evol* 45:643–662.

Kakehashi R, Kurabayashi A, Oumi S, Katsuren S, Hoso M, Sumida M. 2013. Mitochondrial genomes of Japanese *Babina* frogs (Ranidae, Anura): unique gene arrangements and the phylogenetic position of genus *Babina*. *Genes & Genetic Systems* 88:59–67.

Li Z, Yu G, Rao D, Yang J. 2012. Phylogeography and Demographic History of *Babina pleuraden* (Anura, Ranidae) in Southwestern China. *PLoS ONE* 7:e34013.

Li D, Song S, Chen T, Zhang C, Chang C. 2015. Complete mitochondrial genome of the desert toad-headed agama, *Phrynocephalus przewalskii* (Reptilia, Squamata, Agamidae), a novel gene organization in vertebrate mtDNA. *Mitochondr DNA* 26:696–697.

Macey JR, Schulte JA, Larson A, Papenfuss TJ. 1998. Tandem duplication via light-strand synthesis may provide a precursor for mitochondrial genomic rearrangement. *Mol Biol Evol* 15:71–75.

Macey JR, Papenfuss TJ, Kuehl JV, Fourcade HM, Boore JL. 2004. Phylogenetic relationships among amphisbaenian reptiles based on complete mitochondrial genomic sequences. *Mol Phylogenet Evol* 33:22–31.

Song S, Li D, Zhang C, Jiang K, Zhang D, Chang C. 2016. The complete mitochondrial genome of the color changeable toad-headed agama, *Phrynocephalus versicolor* (Reptilia, Squamata, Agamidae). *Mitochondr DNA* 27:1121–1122.

Xia Y, Zheng YC, Miura I, Wong PB, Murphy RW, Zeng XM. 2014. The evolution of mitochondrial genomes in modern frogs (Neobatrachia): nonadaptive evolution of mitochondrial genome reorganization. *BMC Genomics* 15:691.
